# Organic photovoltaic microburritos for photo(electro)catalytic peroxide generation

**DOI:** 10.1039/d5cc06012e

**Published:** 2025-12-11

**Authors:** Anna Tvrdoňová, Marie Jakešová, Jiří Ehlich, Eric Daniel Głowacki

**Affiliations:** a Bioelectronics Materials and Devices Laboratory, Central European Institute of Technology CEITEC, Brno University of Technology Purkyňova 123 61200 Brno Czech Republic glowacki@vutbr.cz

## Abstract

We present organic semiconductor “microburritos” as a scalable microdevice for spatiotemporally precise hydrogen peroxide generation for *in vitro* biological experiments. By integrating an organic donor–acceptor heterojunction with gold, these devices harness light to drive oxygen reduction while simultaneously mediating oxidation of donor molecules in the surrounding medium. We critically compare peroxide generation in different media and characterize the effects of illumination pulsing frequency on peroxide accumulation and the side effect of photothermal heating. The result of our effort is both the establishment of a reliable, light-responsive microdevice and a contribution to understanding the semiconductor-mediated redox cycle, paving the way for advanced bioelectronic and photo(electro)catalytic applications.

## Abstract 2

We report light-responsive gold-organic semiconductor microburritos that drive the photoelectrocatalytic redox cycle through oxygen reduction and donor oxidation, enabling spatiotemporally precise H_2_O_2_ delivery.

## Graphical abstract

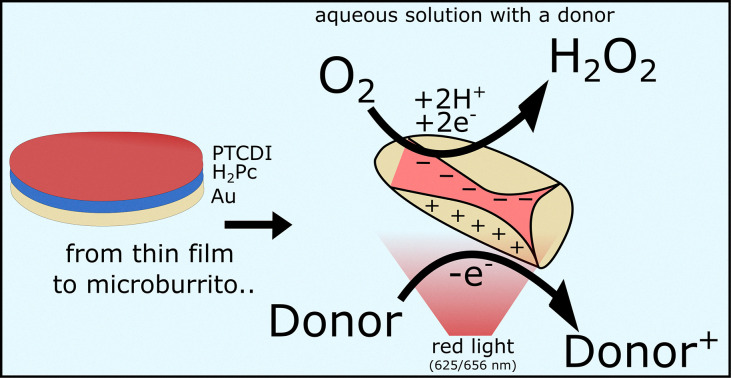

Cellular function relies on the ability to maintain redox homeostasis, a dynamic balance between the generation and elimination of reactive oxygen species (ROS).^1,2^ ROS, once regarded as harmful byproducts, are now recognized as critical signaling molecules that regulate fundamental biological processes. Mitochondrial respiration, NADPH oxidase activity, and other enzymatic pathways continuously generate ROS, among which hydrogen peroxide (H_2_O_2_) has emerged as particularly important. Its relative stability, capacity to permeate membranes, and role as a second messenger allow H_2_O_2_ to influence pathways involved in metabolism, immune response, and programmed cell death.^3–5^ The dual nature of H_2_O_2_, serving as both a physiological regulator and a mediator of oxidative stress, has positioned it as a central focus of redox biology. Experimental addition of H_2_O_2_ remains a cumbersome task in the field of redox biology. Conventional methods to modulate H_2_O_2_ levels *in vitro* include bolus addition,^6^ enzymatic generation,^7^ and redox cycling compounds.^8^ While effective for some applications, these approaches generally lack spatial and temporal control, leading to uniform or poorly defined ROS/H_2_O_2_ exposure. Moreover, biological media rapidly clear peroxide *via* both enzymatic and nonenzymatic pathways. Such limitations hinder the study of localized redox signaling events and complicate the interpretation of biological outcomes.

Semiconductor-based micro- and nanoparticles have emerged as promising alternatives, enabling the generation of ROS/H_2_O_2_ directly at the cellular target. Semiconductors can be triggered by light. The design can be tailored to interface with cells, tissues, or even target subcellular compartments. Inorganic semiconductors such as TiO_2_^9^ and ZnO^10^ nanoparticles are well studied, but rely on UV activation, which limits biological applicability. Silicon nanowires (SiNWs) absorb across the red to NIR spectrum and are biodegradable, making them attractive for transient biointerfaces.^11–13^ When decorated with nanoclustered gold, SiNWs produce up to 5 µM H_2_O_2_ concentration within 15–30 min in PBS without sacrificial reagents due to auto-oxidation. In contrast to inorganic semiconductors, organic materials represent another route. Carbon nitride nanostructures generate mM concentrations of H_2_O_2_ under illumination for 1h, yet their long-term stability remains unclear.^14,15^ The most extensively deployed in biological experiments are organic semiconductor nanoparticles from poly(3-hexylthiophene) (P3HT).^16–18^ Upon visible-light illumination, P3HT demonstrably increases intracellular ROS levels. While clearly experimentally useful, the ROS produced arise mainly from auto-oxidation rather than photo(electro)catalytic H_2_O_2_ production.^19^ This limits total experimental time, and the effect of P3HT oxidation products is not clear. Overall, in the field of semiconductor particles for H_2_O_2_/ROS modulation, the underlying mechanisms are poorly understood, and optimal design principles for stable, scalable devices remain unexplored.

The present study addresses this gap by designing and fabricating organic semiconductor-gold microstructures (“microburritos”) for photo(electro)catalytic H_2_O_2_ generation. In our previous work, we established organic semiconductor heterojunctions as efficient photoelectrodes,^20–22^ where these thin-film PN heterojunctions generate both photocurrent and photovoltage while supporting a selective two-electron oxygen reduction to H_2_O_2_. To validate that the same fundamental charge-generation mechanism operates in microburritos, we fabricated an analogous planar architecture and recorded its transient photocurrent and photovoltage (Fig. 1a). The photoresponses confirm charge separation under red light, consistent with the photovoltaic mode of operation that subsequently drives the photo(electro)catalytic redox cycle. Building on these findings, we present a free-floating microparticle configuration. The fabrication proceeds through sequential deposition of organic semiconductors on a thin gold film, yielding the trilayer (Au/PN), which constitutes the functional core of the device (Fig. 1b). Upon release from the substrate, the free-floating structures spontaneously roll up into a burrito shape, exposing the gold surface to the surrounding medium (Fig. 1c).

**Fig. 1:**
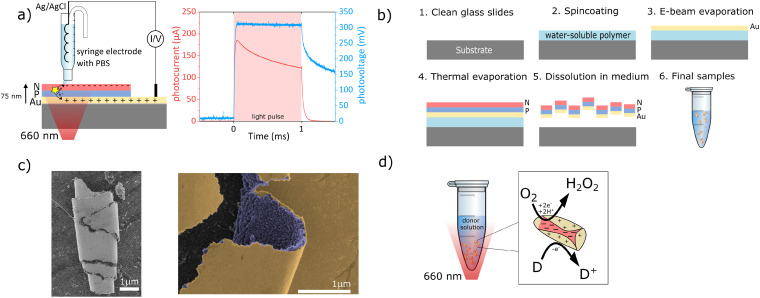
Operation and fabrication of Au/PN microburritos (a) schematic of planar Au/PN heterojunction used to confirm photovoltaic behavior, with corresponding transient photocurrent and photovoltage charactericts under red-light excitation, (b) microburrito fabrication scheme (specified in SI), (c) SEM images of individual rolled microburrito and false-colored close up of the edge of a microburrito showing clear distinction between the gold layer (yellow) and nanocrystalline organic bilayer (purple), (d) photo(electro)catalytic cycle involving charge separation, oxygen reduction reaction, and donor oxidation.

The organic semiconductors, phthalocyanine (H_2_Pc) and *N*,*N*′-dimethylperylenetetracarboxylic diimide (PTCDI) were chosen as the donor–acceptor pair due to their efficient light absorption, charge separation, and stability in aqueous media.^23,24^ H_2_Pc (p-type) strongly absorbs in the visible region (600–700 nm), while PTCDI (n-type) acts as an efficient electron acceptor, transferring photogenerated electrons to molecular oxygen, initiating peroxide formation *via* oxygen reduction reactions (ORRs). The thin layer of gold plays a dual role: it acts as a hole-collecting electrode for the organic bilayer and simultaneously as a catalyst for the oxidation reaction. While other metals, such as Ag, Pd, or Pt, are known catalysts,^25^ they face limitations in the context of biological applications. Pt and Pd decompose H_2_O_2_, whereas Ag suffers from poor stability and dissolution under physiological conditions. In our architecture, the thin gold layer also serves as a semi-transparent electrode, permitting efficient light transmission while simultaneously catalyzing oxidation of the donor molecules in the medium (*e.g.*, glucose,^26^ lactic acid^15^).

The photo(electro)catalytic cycle can be described as follows: upon red-light exposure, excitons (electron–hole pairs) are generated in H_2_Pc and dissociate at the H_2_Pc/PTCDI interface. Photogenerated electrons are transferred to the PTCDI layer, where oxygen reduction results in peroxide.^21^ The complementary holes are collected at the underlying gold layer,^27^ where the oxidation of a donor molecule in the medium occurs, thereby sustaining charge neutrality. The cooperative interplay between the PTCDI as an ORR electrocatalyst,^28^ the organic heterojunction to provide voltage, gold catalyst for oxidation, and medium donor establishes a photo(electro)catalytic redox cycle (Fig. 1c).

To evaluate the photo(electro)catalytic activity of microburritos, we built two custom LED setups (625 nm and 656 nm, respectively) that enable irradiance control and continuous/pulsed protocols. To identify efficient electron donors, we compared three biologically relevant molecules in PBS: 4-(2-hydroxyethyl)-1-piperazineethanesulfonic acid (HEPES), glucose, and sodium formate (SF) under continuous illumination (Fig. 2a). Among these, HEPES supported the highest H_2_O_2_ yield, approximately 150 µM H_2_O_2_ concentration after 1h red light exposure. The strong response with HEPES reflects its facile oxidation. Glucose yielded minimal peroxide, likely due to passivation of the gold surface by its oxidation products, while SF showed limited activity as well. Therefore, the saline salt solution with HEPES (composition specified in SI) was used for all the following experiments, unless otherwise specified. These findings highlight the essential role of medium composition, as the presence of a donor is required to close the redox cycle; without it, no photo(electro)catalytic H_2_O_2_ is produced. This distinguishes our system from ROS generation by polymer nanoparticles such as P3HT, which rely on auto-oxidation.

**Fig. 2:**
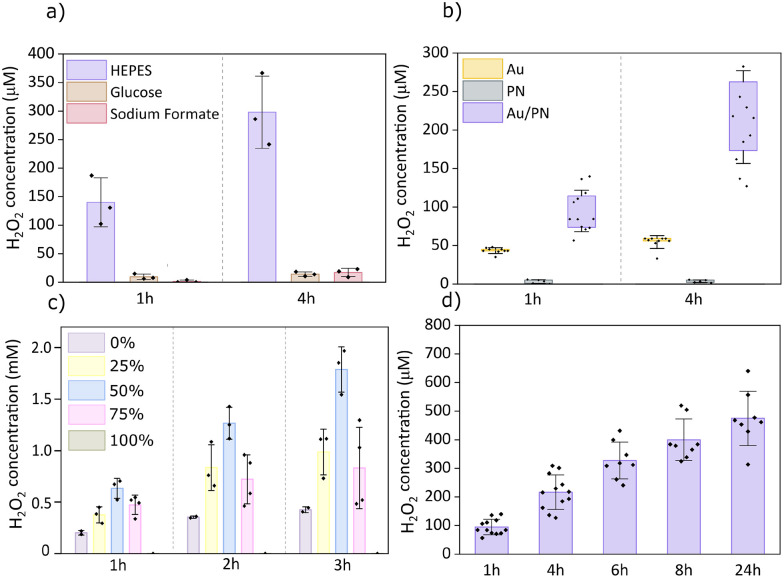
Photo(electro)catalytic H_2_O_2_ generation (a) donor dependence in 1.5 mL PBS with 200 mm^2^ particle area (*n* = 3), (b) control experiments showing necessity of both Au and PN 1.5 mL of salt solution (HEPES as donor) with 200 mm^2^ particle area (*n* = 5–12), (c) varying PN surface coverage on 707 mm^2^ Au/PN planar samples in 2 mL salt solution (HEPES as donor) (0–100%) (*n* = 2–4), (d) Long-term stability of microburritos under continuous illumination in 1.5 mL of salt solution (HEPES as donor) with 200 mm^2^ particle area (*n* = 8–12).

To confirm the necessity of both components (organics and gold), we compared full Au/PN microburritos with controls containing only the gold or photoactive PN bilayer (Fig. 2b). In Au-microburritos, limited peroxide formation can arise from the favorable reaction of donor and oxygen, catalyzed at the gold surface, whereas PN-microburritos generate only trace amounts *via* auto-oxidation of organic semiconductors. To further explore this requirement of both components, we performed experiments with planar samples on PET substrates, where we varied the areal ratio Au : PN (Fig. 2c). The 50 : 50 configuration yielded the highest H_2_O_2_ production, indicating that this balanced composition provides an optimum between charge collection and donor oxidation (Au layer) and surface area for efficient O_2_ reduction (PTCDI layer). The intermediate ratios 75 : 25 or 25 : 75 exhibited comparable efficiency to each other, but lower than 50 : 50. The Au-only (100 : 0) catalyzes spontaneous donor oxidation by oxygen and produces limited peroxide. The PN-only (0 : 100) produces just trace H_2_O_2_. These results underscore the need for a balance of both reductive and oxidative redox processes for optimal photoelectrochemical peroxide production. Overall, it should be noted that the peroxide concentrations reached by the planar samples were higher than the microburritos, despite the total surface area being the same in both experiments. This is likely due to the fact that part of the surface rolled up into the center of the microburrito has low activity. Additionally, in the planar configuration, the Au layer is thinner (Cr/Au 1/9 nm) than in microburritos (15 nm), which improves optical transmission, thereby enhancing the effective light absorption.

Microburritos maintained steady activity, generating H_2_O_2_ concentrations in the hundreds of micromolar range without loss of performance over 24 hours of continuous illumination (Fig. 2d). Such long-term operation is rarely reported for inorganic and organic photo(electro)catalysts, underscoring the robustness of this Au/PN architecture.

Illumination intensity strongly influenced performance, with H_2_O_2_ production scaling with intensity up to 80 mW cm^−2^, beyond which saturation occurred (Fig. 3a). This plateau may reflect peroxide decomposition, as elevated concentrations promote further peroxide reduction to water. At 110 mW cm^−2^, the temperature exceeded 40 °C, cautioning that hyperthermia effects could occur during *in vitro* experiments under extended exposure.^29^ To mitigate thermal effects, pulsed illumination (1 ms ON/100 ms OFF) was applied at 50 mW cm^−2^ (Fig. 3b). Pulsed protocols reduced thermal load but also lowered yields relative to continuous light. Importantly, pulsed illumination may offer a practical strategy in biological experiments where limiting thermal load is critical, even if total peroxide yields are lower than under continuous light. Next, increasing particle concentration (expressed as area of the original sample from 13 to 26 mm^2^ per 100 µL) enhanced H_2_O_2_ generation, but further increase to 100 mm^2^ per 100 µL reduced performance, most notably in the pulsed regime (Fig. 3c), suggesting a diffusion limitation of oxygen and/or donor molecules is reached. The 26 mm^2^ condition yielded a concentration of ∼100 µM H_2_O_2_ after 1 h of pulsed illumination and was selected as optimal without encountering diffusion bottlenecks. In PBS or Salt solution with HEPES, hundreds of µM peroxide was readily produced, whereas in cell culture medium (MEM, supplemented with/without 10% heat-inactivated fetal bovine serum (HI FBS), 1% penicillin–streptomycin), the measured bulk concentration was under 5 µM (Fig. 3d). This outcome cannot be taken to mean that H_2_O_2_ is not produced; rather, both surface biofouling, caused by the accumulation of biomolecules on the particle surface, and/or efficient scavenging by medium components contribute to the lower measurable peroxide levels. Peroxide is likely generated locally in the vicinity of the microburritos but rapidly consumed before diffusing into the bulk, preventing its bulk accumulation. Under these conditions, higher particle loading, 100 mm^2^ per 100 µL, partly offsets this rapid consumption. These results highlight the challenge of operating in complex biological environments.

**Fig. 3:**
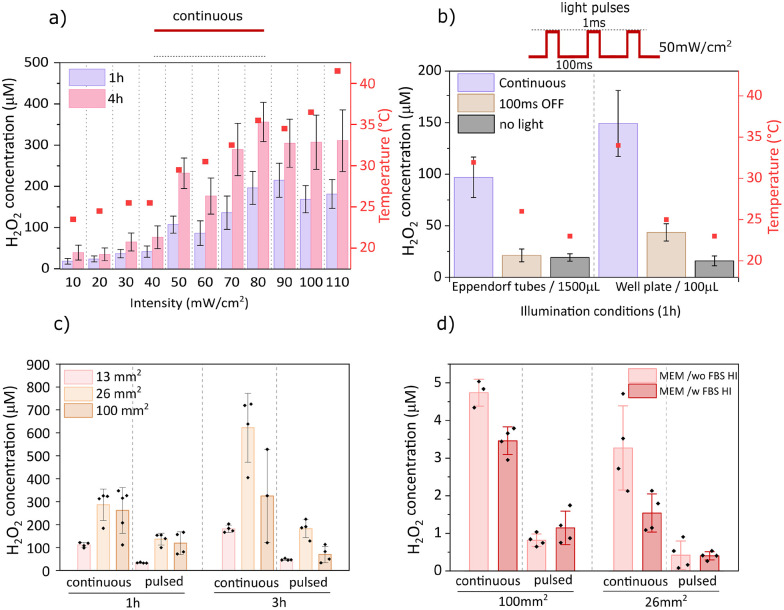
Illumination and environmental effects on photo(electro)catalytic H_2_O_2_ generation (a) dependence on light intensity and measured temperature (after 1h) in 1.5 mL salt solution (HEPES as donor) with 200 mm^2^ particle area (*n* = 8–13), (b) comparison of pulsed and continuous illumination (50 mW cm^−2^) in 1.5 mL/100 µL salt solution (HEPES as donor) with 200 mm^2^/13 mm^2^ particle area (*n* = 6–12), (c) effect of total particle area in 100 µL salt solution (HEPES as donor) under continuous illumination (50 mW cm^−2^) or pulsed light (50 mW cm^−2^, 1 ms ON/100 ms OFF) (*n* = 4–5), (d) H_2_O_2_ concentration measured in cell culture medium (MEM w/wo 10% HI FBS, 1% PS, HEPES as donor) in 100 µL final volume with 100 mm^2^ or 26 mm^2^ particle area under continuous illumination (50 mW cm^−2^) or pulsed light (50 mW cm^−2^, 1 ms ON/100 ms OFF) (*n* = 3–4).

In summary, we have demonstrated that Au/PN microburritos provide a robust and scalable platform for localized photo(electro)catalytic H_2_O_2_ generation under red-light illumination. The devices operate *via* a cooperative redox cycle, in which excitons generated at the PN heterojunction separate into holes and electrons. The photogenerated electrons drive oxygen reduction on the organic semiconductor surface, while the gold layer collects photogenerated holes and catalyzes donor oxidation to sustain the redox cycle. The donor screening confirmed that HEPES enabled efficient H_2_O_2_ generation, whereas glucose or formate supported lower activity, underlying the critical importance of medium composition in governing photo(electro)catalytic output. However, HEPES does not behave as an inert additive; following oxidation, it forms radical intermediates. We recently recognized that oxidized HEPES byproducts may be a source of cytotoxicity.^30^ Consequently, while HEPES can enhance the photo(electro)catalytic yield, its use in biological contexts must be carefully evaluated. Significant H_2_O_2_ production occurred only in the complete system when both components were present. The devices exhibit longer-term stability, tunable output with illumination protocols, and selective H_2_O_2_ generation; however, performance is challenging to evaluate and quantify in complex cell culture media. Compared to other available approaches (specified in SI), microburritos offer a combination of stability and sustained H_2_O_2_ production. Conjugated polymer nanoparticles, such as P3HT nanoparticles, yield up to 1 mM H_2_O_2_ in PBS under white light illumination; however, their reliance on auto-oxidation limits experimental utility.^31^ Silicon nanowires decorated with gold also reach low µM H_2_O_2_ levels in PBS under white light, but also at the cost of auto-oxidation. In contrast, microburritos reproducibly generate hundreds of µM peroxide concentration in PBS or Salt solution with HEPES under red light. Red light produces less spurious biophysical effects compared with higher-energy light, and would be critical for potential *in vivo* applications. Our tests show that microburritos maintain steady performance for at least 24 hours. Moreover, we have detected low µM H_2_O_2_ levels in cell culture medium, despite scavenging. Importantly, preliminary cell-viability tests (Fig. S8) further show that microburritos are well tolerated by cells. Therefore, we believe microburritos are a competitive choice for *in vitro* cellular experiments where spatiotemporally precise and reliable peroxide delivery is desired. Our study shows limitations of this approach, however. The main limitation is the necessity of supplying a donor, in this case, HEPES. We have not identified a suitable alternative. The effects of oxidation products are also a potential issue for biological experiments. Future experiments should focus on increasing the photovoltage of these devices, to be able to drive a larger range of oxidation reactions, and modifying the back-contact from gold to other catalytic metals which may enable the oxidation of other donor molecules.

This work was supported by the European Research Council (ERC No. 949191), and funding from the Czech Science Foundation GAČR (grant agreement No. 25-18184X). The authors acknowledge CzechNanoLab Research Infrastructure supported by MEYS CR (LM2023051) and Brno University of Technology under project CEITEC VUT-J-24-8616. The authors also gratefully acknowledge the Sabine Erschen, Linda Waldherr, and Rainer Schindl from the Division of Medical Physics and Biophysics Medical University of Graz, Austria, for their support and the use of their laboratory facilities.

## Conflicts of interest

There are no conflicts to declare.

## Supplementary Material

CC-062-D5CC06012E-s001

## Data Availability

All data used in this paper can be downloaded at https://figshare.com/s/381cc378729c93de898c or obtained from the corresponding author. The data supporting this article have been included as part of the supplementary information (SI). Supplementary information is available. See DOI: https://doi.org/10.1039/d5cc06012e.
