# Supplementary material for: Organic photovoltaic microburritos for photo(electro)catalytic peroxide generation
Source: Chem Commun (Camb). 2025 Dec 11;62(4):1293–7. doi: 10.1039/d5cc06012e (PMC12710207; doi:10.1039/d5cc06012e)
Supplement: CC-062-D5CC06012E-s001 [file CC-062-D5CC06012E-s001.pdf]

Supplementary Information (SI) for ChemComm.
This journal is © The Royal Society of Chemistry 2025

Electronic supplementary information (ESI) for:

Organic photovoltaic microburritos for photo(electro)catalytic peroxide generation

Anna Tvrdoňová, Marie Jakešová, Jiří Ehlich, and Eric Daniel Głowacki*

Experimental methods:

Materials:

Polyvinylalcohol - PVA, Mw 31 000 g/mol (Microchemicals).

Phthalocyanine - H_2Pc (from TCI) and *N,N′*-dimethylperylenetetracarboxylic diimide – PTCDI (from BASF) purified by 3× temperature-gradient sublimation.

Phosphate Buffer Saline (Roth)– NaCl 8 mM, KCl 0.2 mM, Na_2HPO_4 1.4 mM, KH_2PO_4 0.3 mM mixed with donor in the final 10mM concentration – Glucose (Roth), Sodium Formate (Lachema), HEPES (Sigma Aldrich).

Salt solution - NaCl 140 mM, $CaCl_2$ 2.5 mM, $MgCl_2$ 2 mM, HEPES 10 mM (Sigma Aldrich), Glucose 20 mM (Roth), KOH 3 mM, NaOH 2 mM, pH = 7.4.

Cell Culture Medium – MEM (Pan BioTech) - Eagle's salts, 2.2 g/L $NaHCO_3$, L-glutamine, 25mM HEPES, supplemented with 10% heat-inactivated Fetal Bovine Serum (Gibco) and antibiotics penicillin/streptomycin (Gibco).

Microburritos Fabrication:

Microburritos were fabricated using a physical vapor deposition processes on clean microscope slide glass. After solvent cleaning (10 min each of the solvents/solution – acetone, isopropyl alcohol, 2% hellmanex (detergent), deionized water), the glass slides were treated with oxygen plasma (5 min, 4 sccm O_2, power 100% using Resist Stripper Diener electronic NANO plasma cleaner), and a layer of 10% PVA was spin-coated at 1500 rpm using Laurell WS-650-23 B spin coater, and subsequently baked at 80 °C for 10 min. Next, a 15 nm gold layer was evaporated using electron beam evaporator BESTEC under a base pressure of $< 1 \times 10^{-6}$ mbar at a rate of 1 $Å \cdot s^{-1}$. Subsequently, semiconductor layers (H_2Pc/PTCDI) were deposited using Edwards 306 Thermal Evaporator at a rate of 3-6 $Å \cdot s^{-1}$ for both materials at a base pressure of $< 1 \times 10^{-6}$ mTorr, forming a total thickness of 60 nm (30 nm each of p-type and n-type). The final sample consists of a three-layer stack designed to detach upon the dissolution of the PVA layer in an aqueous medium (PBS + donors / Salt Solution). To facilitate handling, the coated glass slides were cut into 25 × 8 mm pieces and placed into 2 mL Eppendorf tubes containing the medium. To promote film detachment, the samples were exposed to ultrasound for 20 min. Upon detachment, the film spontaneously rolls up, forming microburritos. The final concentration of microburritos was determined by the total area of the processed glass pieces.

Planar devices Fabrication:

Planar devices were fabricated using physical vapor deposition processes on clean PET foil substrates (30 mm diameter) (*Fig. 2*). After solvent cleaning (10 min each of the solvents/solution – isopropyl alcohol, deionized water), the substrates were treated with oxygen plasma (1 min, 4 sccm O_2, power 50% using Resist Stripper Diener electronic NANO plasma cleaner). Next, a 1 nm chromium (Cr) adhesion layer and 9 nm gold (Au) layer were deposited using an electron beam evaporator BESTEC under a base pressure of < 1×10^{-6} mbar at a rate of 0.2 $Å \cdot s^{-1}$ for Cr and 1 $Å \cdot s^{-1}$ for Au. Right after, the samples were covered by a shadow mask from aluminum foil, which covered each sample accordingly to achieve different area ratios between the bottom (Cr/Au) layer and the photoactive (PN) layer, as shown in Fig. 2. Subsequently, semiconductor layers (H_2Pc/PTCDI) were deposited using Edwards 306 Thermal Evaporator at a rate of 3-6 $Å \cdot s^{-1}$ for both materials at a base pressure of < 1×10^{-6} mTorr, forming a total thickness of 60 nm (30 nm each of p-type and n-type).

Illumination protocols:

Two custom setups - the first was based on a Farnell (656 nm) high-power LED, employed for H_2O_2 photoproduction using an Eppendorf tube setup. The LED was operated using a Thorlabs LED Driver (DC2200), which allowed for adjustment of light intensity in the range of 10-110 mW/cm² and enabled both continuous and pulsed illumination protocols. The second light source was made of Wurth Elektronik LEDs (625 nm), integrated into a custom setup designed for a 96-well plate. This system used an LED Driver LDD 1500L controlled by an Arduino Nano microcontroller. The light intensity could be set between 50-100 mW/cm². Light intensities were measured using a calibrated Thorlabs Si p-i-n diode (Thorlabs SM1PD1B). The emission spectra of both LEDs were specified by the manufacturer and independently verified using a fiber spectrometer *(Fig. 3)*.

Quantification of H_2O_2:

The concentration of H_2O_2 was quantified spectrophotometrically. In our lab, we use tetramethyl benzidine, TMB, which gets oxidized, forming a blue-colored product measured via absorbance at 653 nm using PlateReader BioTek Synergy H1, in 96-well flat-bottom plates. The oxidation reaction is catalyzed by the horseradish peroxidase enzyme, HRP, and conducted in the citric acid-phosphate buffer solution. The aliquots taken from the samples (10-40 μL) were mixed with the corresponding volume of TMB/HRP/buffer solution to a final 200 μL of the solution. The absorbance values were recalculated to concentration values based on calibration curve (*Fig. 4*).

For quantification in cell culture media, we use Amplex UltraRed Reagent. The reagent is a fluorogenic substrate for horseradish peroxidase that reacts with H_2O_2 in 1:1 stoichiometric ratio to produce Amplex UltraRed, a brightly fluorescent and strongly absorbing reaction product. We measured the fluorescence at excitation/emission maxima 530/590 nm using PlateReader BioTek Synergy H1, in 96-well flat-bottom black plates. 25 μL aliquots taken from the sample were mixed with 25 μL of deionized water to a final volume of 50 μL, and afterwards mixed with 50 μL of the Amplex UltraRed/HRP working solution in citric acid-phosphate buffer. The fluorescence values were recalculated to concentration values based on a calibration curve (*Fig. 5*).

SEM imaging:

High-resolution imaging was performed using a Scanning Electron Microscope FEI Verios 460L. Imaging was conducted at low acceleration voltages (< 3 kV), current 6.3pA, TLD detector, minimizing sample charging and beam-induced damage while enhancing surface contrast.

Cell Viability Assay:

The human melanoma cell line A375 (provided by the Medical University of Graz cell bank) was cultured at 37°C and 5 % CO_2 in Dulbecco's Modified Eagle's Medium – high glucose (DMEM, Sigma-Aldrich) supplemented with 10% Fetal Bovine Serum. Cells were cultured in tissue culture flasks, media was changed every 3 days, and splitting was performed at 80% confluency. For passages, cells were dissociated with TrypLE (Gibco) and subsequently counted using the LUNA-II™ automated cell counter (Logos Biosystems). For the microburritos treatment, 20,000 cells in 100 μL FluorobriteTM (Gibco) supplemented with 10% FBS per well were seeded into flat-bottom, blank 96-well plates. 24 hours after seeding, the culture medium was replaced with medium supplemented with microburritos (13,26,100 mm^2 per 100 μL) and left for 24 hours. Cell Viability analysis was evaluated using the PrestoBlue Assay following the manufacturer's guidelines to determine cell viability. Growth controls without microburritos were included, and blank wells containing medium with the corresponding microburrito concentration (but no cells) were measured to account for background fluorescence originating from the microburritos themselves. Fluorescence signals were detected on a CLARIOstar Plus plate reader (Ex 540-570 nm, Em 580-610 nm).

Electro(Photo)Response (EPR):

Electro(Photo)Response (EPR) is used for the determination of maximum photovoltage and photocurrent. EPR measurements were performed using a 15-bit, two-channel PicoScope 5243B oscilloscope. All measurements were carried out in the dark. Optical excitation was provided by a 660 nm red LED (Thorlabs M660L4, 1.2 mW/mm^2) positioned beneath the sample stage, behind a transparent glass window. Electrical contact was made with an AgCl-coated silver wire inserted into a syringe filled with PBS. The syringe was mounted on a holder with a screw-controlled z-axis to allow precise positioning above the device. Electrolyte contact was formed without mechanical touching by forming a droplet at the syringe tip: the droplet height was controlled manually via an empty syringe connected by flexible tubing. The area of the electrode interface was approximately 12.57 mm^2. Photovoltage signals were recorded directly with the oscilloscope. Photocurrents were obtained using a low-impedance (50 Ω) low-noise current amplifier (FEMTO DLPCA-200).

Figures and Tables:

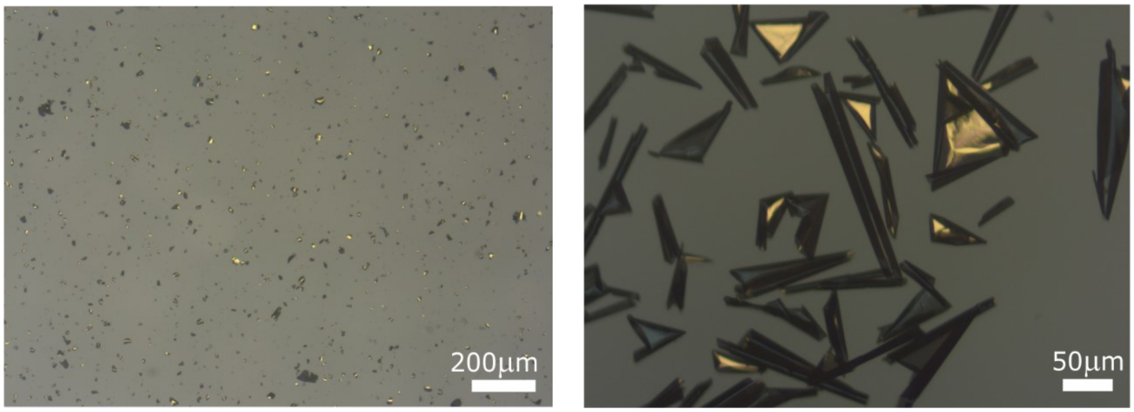

Fig. 1: Optical microscopy images of Au/PN microburritos: left image shows dispersed rolled structures obtained after ultrasonication, and right image shows microburritos prepared without ultrasound

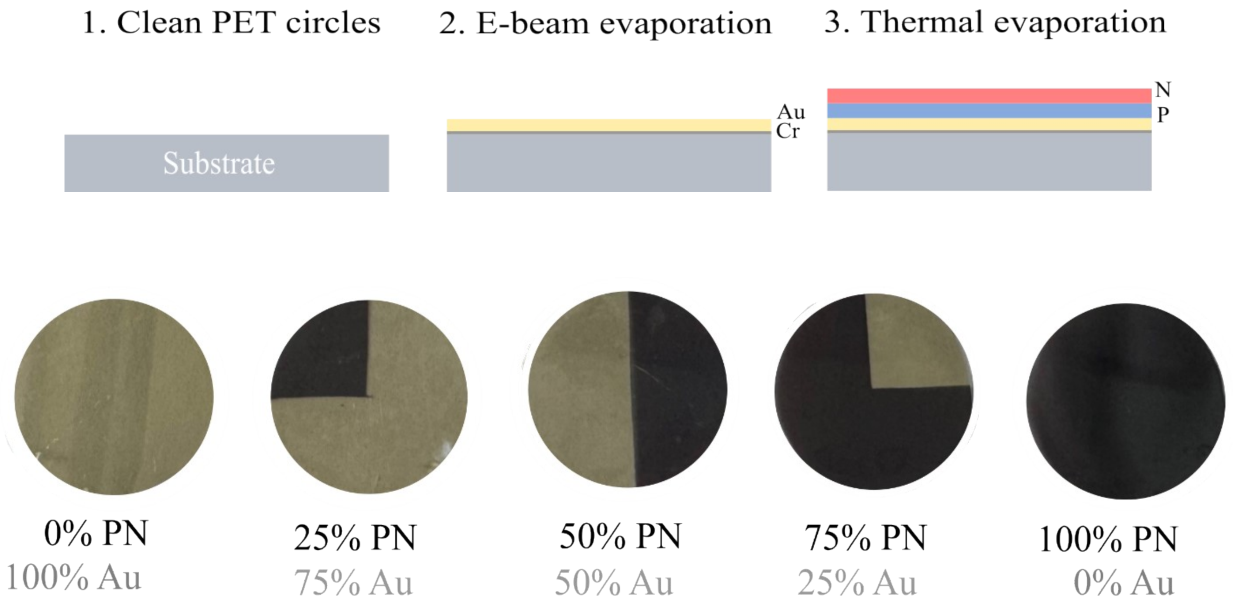

Fig. 2: Fabrication process of the planar devices, showing possible designs for different Au/PN surface ratios

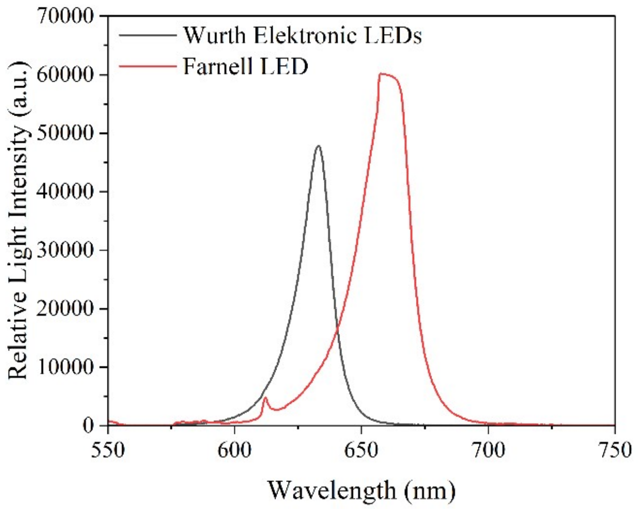

Fig. 3: Emission spectra of both LEDs used in this study

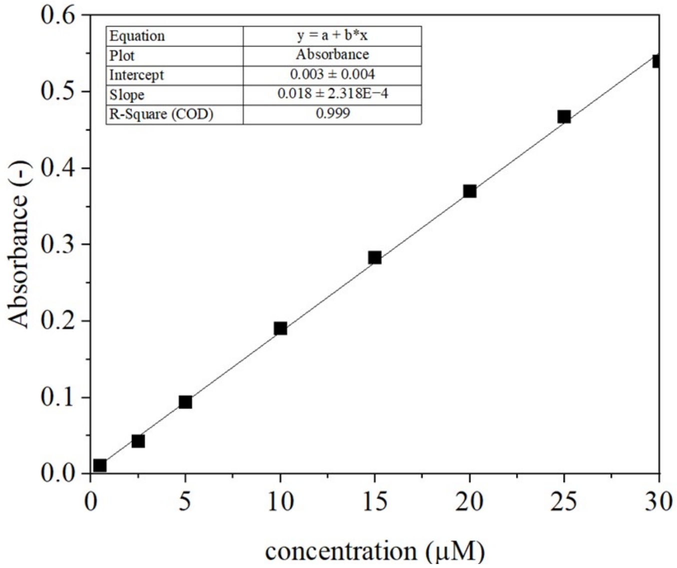

Fig. 4: Calibration curve for H_2O_2 determination by TMB absorption assay

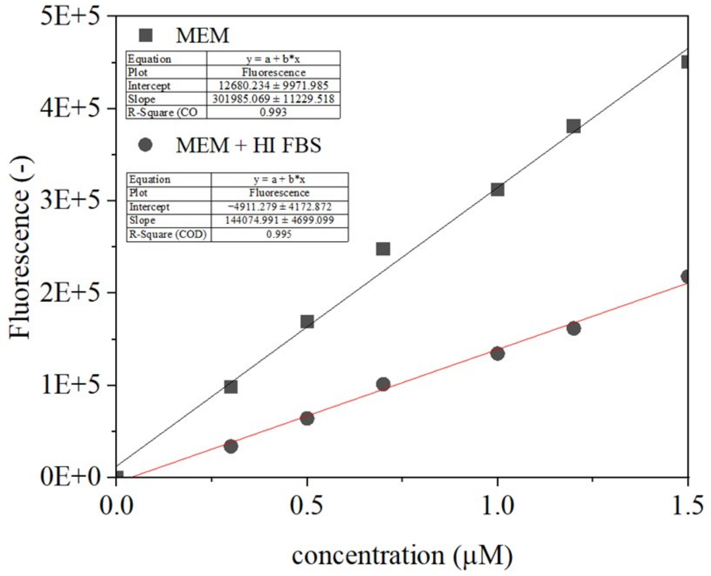

Fig. 5: Calibration curve for H_2O_2 determination in MEM and MEM+HI FBS by Amplex Red Assay

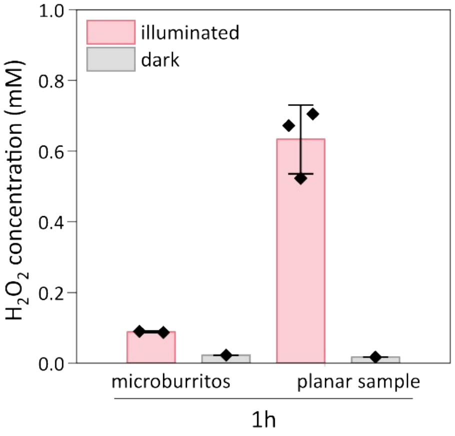

Fig. 6: Comparison of microburritos vs. planar devices (1:1 Au:PN) in 2 ml salt solution (HEPES as donor) under continuous illumination (1h, 50 mW/cm²). The two types of samples have the same total area. This result shows that planar morphology produces higher peroxide "bulk" concentrations overall. This is due to an interplay of two factors caused by microburrito rolling: loss in catalytically-active surface area, which remains in the rolled-up core of the sample; and lower effective light absorption, because of PN material in the rolled-up core being shadowed by overlaying PN/Au layers.

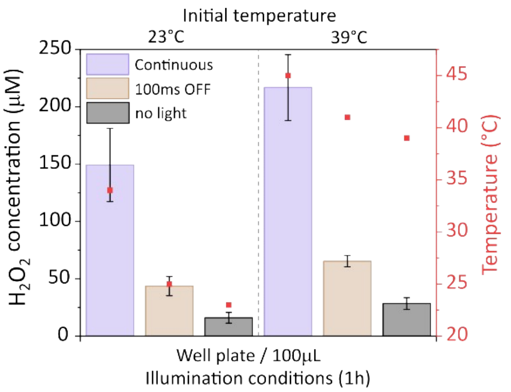

Fig. 7: Comparison of microburritos 13 mm² per 100 µL under pulsed and continuous illumination (1h, 50 mW/cm²) in salt solution (HEPES as donor) when exposed to different initial temperatures: left shows the experiment at 23°C ambient temperature, while the right panel shows the resulting peroxide under illumination inside of an incubator at 39°C. This shows both the effect of light protocol (continuous > pulsed 1ms ON/100ms OFF) and temperature (39°C > 23°C) influencing the final H_2O_2 production.

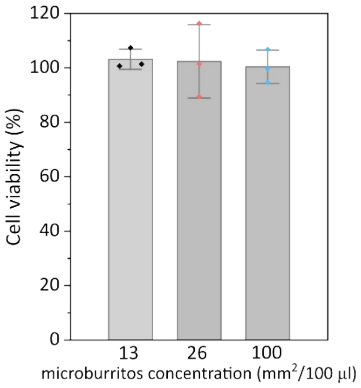

Fig. 8: Cell viability measurements of A375 melanoma cancer cells after incubation with different total areas of burritos for 24h

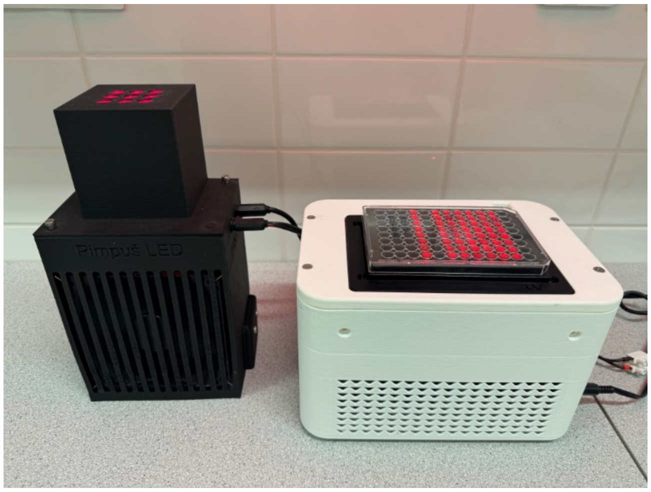

Fig. 9: Custom LED illumination setups (left) configuration for Eppendorf tubes using 656nm LED, (right) configuration for 96-well plates using 625nm LEDs

Table 1: Experimental parameters and measured peroxide concentrations for light-triggered H_2O_2 generation using different photoactive materials.

Material	Particle concentration	Medium	Light	Intensity	Exposure time	peroxide concentration
Au-SiNWs[1]	fixed NWs on area 0.25 x 0.25cm	PBS	white Xe lamp	150-200mW/cm^2	0.25-0.5h	5 µM
P3HT NPs[2]	5 % w/w in PBS	PBS	white LED lamp	2.4mW/cm^2	4h	1400 µM
P3HT thin film general[3]	0.5cm^2	H_2O	white LED lamp	100 mW/cm^2	20 h	99 µM
Au/PN microburritos	200mm^2 per 1500µL	Salt solution + HEPES	red LED	50mW/cm^2	4h	230 µM
Au/PN microburritos	100mm^2 per 100µL	MEM + HEPES	red LED	50mW/cm^2	1h	5 µM

References:

1 A. W. Phillips, R. Parameswaran, E. Lichter, J. Jeong, L. Meng, M. Burke, K. Koehler, Y. V. Lee and B. Tian, *ACS Appl Mater Interfaces*, 2021, **13**, 15490–15500.

2 M. Zangoli, A. Cantelli, A. Candini, A. Lewinska, F. Fardella, A. Tino, G. Tommasini, M. Wnuk, M. Moschetta, S. Perotto, M. Lucarini, C. Tortiglione, G. Lanzani and F. Di Maria, *Journal of Physical Chemistry C*, 2023, **127**, 4672–4683.

3 M. Gryszel, M. Sytnyk, M. Jakešová, G. Romanazzi, R. Gabrielsson, W. Heiss and E. D. Głowacki, *ACS Appl Mater Interfaces*, 2018, **10**, 13253–13257.
